# Transcriptome analysis of the transition from primary to secondary growth of vertical stem in *Eucalyptus grandis*

**DOI:** 10.1186/s12870-024-04731-3

**Published:** 2024-02-08

**Authors:** Fangping Zhou, Haonan Zhang, Shanshan Chen, Chunjie Fan

**Affiliations:** 1 State Key Laboratory of Tree Genetics and Breeding, Chinese Academy of Forestry, Beijing, 100091 China; 2 Key Laboratory of State Forestry Administration On Tropical Forestry, Research Institute of Tropical Forestry, Chinese Academy of Forestry, Guangzhou, 510520 China; 3 College of Life Science, Northeast Forestry University, Harbin, China; 4 State Key Laboratory of Tree Genetics Breeding, Northeast Forestry University, Harbin, China; 5 Yuelushan Laboratory, Central South University of Forestry and Technology, Changsha, Hunan China

**Keywords:** *Eucalyptus grandis*, Secondary growth, Wood formation, Stem

## Abstract

Eucalyptus was one of the most cultivated hardwood species worldwide, with rapid growth, good wood properties and a wide range of adaptability. Eucalyptus stem undergoes primary growth (longitudinal growth) followed by secondary growth (radial growth), which produces biomass that is an important source of energy worldwide. In order to better understand the genetic regulation of secondary growth in *Eucalyptus grandis*, Transcriptome analyses in stem segments along a developmental gradient from the third internode to the eleventh internode of *E. grandis* that spanned primary to secondary growth were carried out. 5,149 genes that were differentially expressed during stem development were identified. Combining the trend analysis by the Mfuzz method and the module-trait correlation analysis by the Weighted Gene Co-expression Network Analysis method, a total of 70 differentially expressed genes (DEGs) selected from 868 DEGs with high connectivity were found to be closely correlated with secondary growth. Results revealed that the differential expression of these DEGs suggests that they may involve in the primary growth or secondary growth. AP1, YAB2 TFs and *EXP* genes are highly expressed in the IN3, whereas NAC, MYB TFs are likely to be important for secondary growth. These results will expand our understanding of the complex molecular and cellular events of secondary growth and provide a foundation for future studies on wood formation in Eucalyptus.

**Supplementary Information:**

The online version contains supplementary material available at 10.1186/s12870-024-04731-3.

## Introduction

Owing to their broad adaptability, rapid growth and multipurpose properties, Eucalyptus had been as the most widely planted hardwoods in the worldwide, which provided renewable resources for the production of pulp, paper, timber and bioenergy [1, 2]. The considerable economic importance of Eucalyptus wood has been a driving force to unravel the genetic regulation mechanisms of wood formation in Eucalyptus as a path to genetic improving wood production and quality. The process that trees develop into wood through many years of growth is always accompanied by cell division and differentiation, secondary wall thickening, and programmed cell death. Meanwhile, plant secondary growth is of great significance to human beings because the secondary xylem is the raw material of wood pulp and paper making, construction industry and textile industry. The plant secondary growth is mainly a series of active processes initiated by the vascular cambium, including vascular tissue formation, secondary cell wall formation, lignification (lignin deposition), programmed cell death (PCD), and heartwood formation, etc. The process of secondary cell wall formation mainly includes the biosynthesis and assembly of four components: polysaccharides (mainly cellulose and hemicellulose), lignin, cell wall proteins and some secondary metabolism such as flavonoids, tannic acid, pectin, etc. In particular, the directional arrangement of cellulose and the deposition of lignin are signature events in the process of secondary cell wall formation [3, 4].

Many studies have been conducted to help understand these developmental processes and wood formation-related genes were selected by using cDNA array technology during tension wood formation [5], xylem vs phloem subtractive library [6], juvenile *versus* mature wood and vice versa subtractive libraries [7], illumina mRNA-Seq technology and digital expression profiling [8], high-density BAC library construction [1], transcriptomes analysis of the developing xylem and leaf [9]. These approaches provided very useful information on *Eucalyptus* wood formation-associated gene expression. And based on these results, several genes were identified which *EgrFAS* affected cellulose deposition [10], *EgrTUB1* associated with wood-fiber formation [11] and EgMUR3 and EgH1.3 performed in lignin biosynthetic pathway, while EgMYB1 is a regulator of SCW formation [12–14]. Meanwhile, transcriptomic analysis in tension wood also selected two most significant TFs *PtrHSFBb3-1* and *PtrMYB092* and then were validated in *Populus trichocarpa* [15]. Recently, RNA-seq analysis in *P. trichocarpa* stems identified 95 VCS (vascular-cambium-specific) TFs which involved in epigenetic modification in cambium cell layers for wood formation and *PtrVCS2* was validated as controlling the expression of *PtrWOX4a* [16]. With a series of validation experiments, the regulatory pathway in which the second most abundant *VCS*, through the system’s. However, there are still numerous wood formation-related genes waiting for uncovering and deciphering.

In *Populus* and herbaceous species Arabidopsis, the transition from primary to secondary growth in stem was used as a unique system to identify processes specific gene to secondary growth [17–19]. In *Populus*, 271 transcript regulatory and structural genes involved in secondary growth and secondary wall biogenesis and 3,000 differentially expressed genes during stem development were characterized by cDNA-AFLP and genomic microarray respectively [18, 19]. But due to technical limitations, these transcripts represent far less than the 28,294 annotated genes that have been found to be expressed during cambial growth and wood formation by RNA-seq methods [20]. Recently, 15,838 differentially expressed transcripts along the shoot developmental gradient from the shoot apex to the fifth internode of *Populus*, of which 1,216 were transcription factors (TFs), were found in combination with PacBio Iso-Seq and RNA-seq analysis [21].

However, Eucalyptus with the characteristics of rapid growth and good-quality wood fiber was not performed until now. The available genome sequence in *E. grandis* provided the opportunity to insight on wood development and identify key genes to secondary growth in Eucalyptus [2]. Hence, in this study, transcriptome sequencing and further differential expressed genes, Mfuzz and WGCNA [22] analysis in various internodes of stem that spanned primary to secondary growth were performed. It would be helpful to unravel the molecular mechanism of the development transition from the primary growth to secondary growth in Eucalyptus. Meanwhile, several transcription factors and genes which involved in primary growth or secondary growth were also identified. Moreover, a hierarchy regulatory network regulating secondary growth was also constructed, which would be applied for further molecular breeding in Eucalyptus.

## Material and methods

### Plant material and culture conditions

Thirty healthy, rapidly growing, six-month-old *E. grandis* trees (Clone GL1) at a nursery in Zhaoqing, Guangdong (China) were selected for the study. The sample was collected on a clear day between 10 and 12 am in June. Following measurement of internode lengths, the internodes (excluding the nodes) from the apical bud to the base of the shoot were excised into liquid nitrogen in the field and stored at -80 °C. RNA isolation from at least three trees was used for next transcriptome sequencing and this experiment was performed three times.

The internodes were harvested and processed for histochemical analysis according to the method described by [18]. A 2 mm long segment in first internode and 5mm long segment in other internodes cut by knife blade were fixed in FAA for microscopy. After dehydration, infiltration and embedding with paraffin, thick Sects. (10 μm) were obtained and stained with toluidine blue-O (Sigma-Aldrich, St. Louis).

### RNA isolation and sequencing

The EASY spin Plus Plant RNA Kit (Aidlab Biotechnologies Co., Ltd., Beijing, China) was used to extract total RNA in accordance with the manufacturer's instructions. The NanoDrop 2,000 spectrophotometer (NanoDrop Technologies, Wilmington, DE, USA) was used to measure the yield and purity of RNA. The extracted RNA concentration was more than 100ng/μL, and RNAs with A260/A280 values between 1.8 and 2.2 and A260/A230 values more than 1.0 were regarded as dependable. After that, the Agilent 2100 Bioanalyzer was used to verify the integrity of the RNA, and samples of RNA with an RNA integrity score (RIN) more than 8 were kept in a refrigerator at -80 °C for later processing.

Using a mRNA-Seq 8 Sample Prep Kit from Illumina, 10 μg of each sample's total RNA was utilised to isolate poly(A) mRNA and create a nondirectional Illumina RNA-Seq library. By dissolving the removed gel slices at room temperature, a modified gel extraction procedure was used. A Bioanalyzer Chip DNA 1000 series II (Agilent) was used for library quality control and quantification. Each library's insert size was 200 bp, and clean raw reads were obtained through sequencing using Illumina HiSeqTM 2000. All clean reads were mapped to the reference genome (*Eucalyptus grandis* genome v2.0, phytozome) following filtering with SOAPnuke (Version 1.4.0).

### Analysis of differentially expressed genes

Using all collected reads per kilo bases per million mapped reads (RPKM) (1.16.1) as a basis, differential expression analysis was carried out using the DESeq2 R package (1.16.1) [23]. An examination of differential expressions between pairs was done. DEGs were calculated using transcripts with log_2_FC > 1 or log_2_FC < -1, and *P*-value < 0.05. The R programming environment was used to create and develop all additional statistical analyses and figures.

### Functional annotation and GO / KEGG analysis

Gene Ontology (GO) enrichment analysis of the collected DEGs was performed using agriGO (version 2.0) (http://systemsbiology.cau.edu.cn/agriGOv2/), and the results were adjusted by *P* value less than 0.05. The enrichment of DEGs in KEGG pathways was tested using the cluster Profiler R programme. Venn diagrams were created with Tbtools programme [24].

### Temporal analysis

To evaluate the expression pattern of differential expression genes (DEGs), a temporal analysis was carried out. Every sample had its expression value normalised to 0, log2 (v1/v0), and log2 (v2/v0). These DEGs were clustered by Mfuzz package of R ([25]) with a maximum unit change in model profiles between time points of 1, maximum output profile number of 12, and minimum ratio for fold changes of DEGs of 2. The clustered profiles (*p* ≤ 0.05) were used for functional annotation analysis through the hypothesis test. Using the hypothesis test, functional annotation analysis was performed on the clustered profiles (*p* < 0.05). For these strongly clustered profiles, the GO keywords or KEGG pathways with a Q-value ≤ 0.05 were deemed considerably enriched.

### Gene co-expression network construction

The R package WGCNA V1.41–1 was used for WGCNA analysis [22]. Genes with minimal expression fluctuation (standard deviation < 0.2) were filtered out, and DEGs were then classified into strongly correlated gene clusters using pairwise correlation analysis based on the expression value of each gene. Co-expression modules were built using the automatic network construction function's block-wise modules. In order to create cluster families within the network, cluster correlation was used. Based on the correlation coefficient (r^2^ > 0.8 and *p* value < 0.001), the modules that were most closely related were chosen. Additionally, the correlation between genes and modules is assessed through the computation of the KME value (module eigengene-based connectivity), which is often assigned to specific hub genes when |KME|≥ 0.8.

Genes having a weight value greater than 0.5 were chosen to execute in order to further determine the hub genes related to Eucalyptus secondary growth. Using the Cytoscape programme (version 3.9.0) (http://www.cytoscape.org/), the network image was plotted.

### Quantitative RT-PCR analysis

The expression of candidate genes was determined using quantitative real-time polymerase chain reaction (qRT-PCR). Plant material was collected as described previously. 20 genes with fold-changes, and falling into various expression patterns, were selected for verification. cDNA synthesis originated from 1.0 μg of total RNA was performed by using Super Script III kit (Invitrogen) according to the manufacturer’s instructions. cDNA aliquots corresponding to equal amounts of RNA were used for the quantification of mRNA by qPCR using the LightCycler 96 real-time quantitative PCR detection system (Roche, Indianapolis, IN, USA). Primers were designed using Primer3 software. The reaction volume was 20 μL, including 2.0 μL of the cDNA template, 0.8 μL of 10 mM Forward primer, 0.8 μL of 10 mM Reverse primer, 6.4 μL of ddH_2_O and 10 μL of SYBR® *Premix Ex Taq*™ (Tli RNaseH Plus, Takara). The PCR reactions were carried out using the following conditions: 95 °C for 30 s, and subsequently 40 cycles of 95 °C for 5 s, 60 °C for 30 s and 95 °C for 15 s. The *EgrEF2* gene was selected as internal reference gene [26]. For every sample, three biological and three technical replicates were used to conduct every reaction. All of the chosen genes' relative expression levels were measured using the 2^−Δ*ΔCt*^ method [27].

## Results

### Eucalyptus stem development and Vertical stem segments represent different developmental stages

**Fig. 1:**
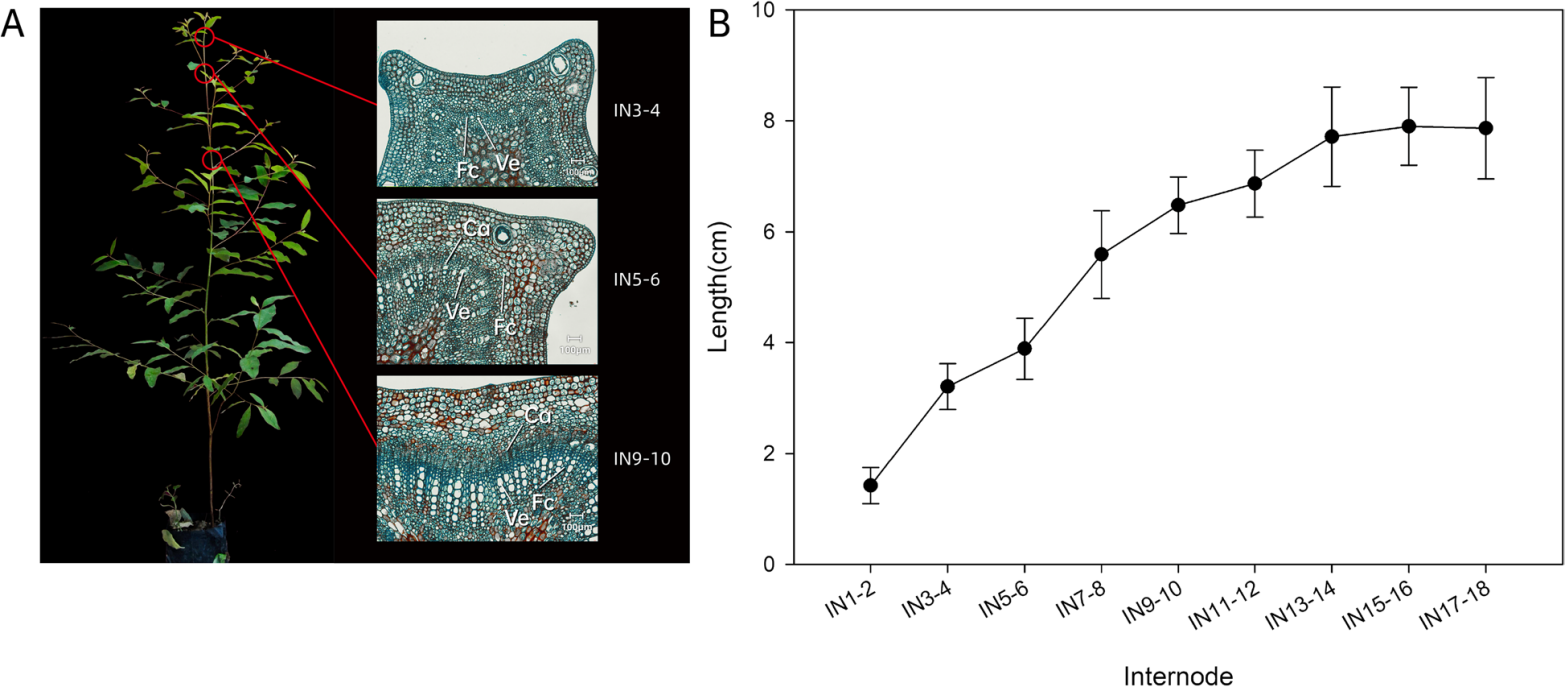
Growth and development of different stem internodes. **A** Anatomy of stem internodes from developing *Eucalyptus grandis* GL1, Schematic showing the regions from the basal stem used for sections in IN3-4, IN5-6, IN9-10, which are Toluidine Blue-O-stained cross sections from stem internodes 3 to 9 of an actively growing young tree. Ca, vascular cambium; Fc, Fiber cell; Ve, Vessel elements. Bars = 100 μm. **B** Apical shoot internode growth of *E. grandis* GL1, elongation zone and change in internodes lengths along the developing stem of the 30 trees sampled

To clarify the changes from primary to secondary growth, the internodes were collected for histological chemistry from the apex to the base of stem. As shown in Fig. 1A, the xylem and phloem were well differentiated between different stem segments. The IN3 stem section is in the primary growth stage, where the vascular bundles composed of primary xylem and phloem form peripheral microtubules on the cross section, and the vascular bundles are separated by interfascicular parenchyma. Therefore, each microtubule cluster is not fully connected to form a complete ring xylem. The IN5 stem section is in the transition node from primary xylem to secondary xylem of stem, and part of parenchyma cells between vascular bundles begin to differentiate into interfascicular cambium at this stage. While the IN9 stem section is the secondary growth stage with obvious secondary growth, and the cambium cells differentiate into secondary xylem and secondary phloem, which has obviously lignified secondary walls and phloem fibers. Meanwhile, the length of internodes varies measured thirty plants from apex to the base further verify the changes from primary to secondary growth (Fig. 1B). From IN1 to IN6, the growth mainly involved the elongation of stem. And then the elongation of internode slowly increased from IN7 to IN12. Next, the length of internodes from IN13 to the base showed only slight increases while the growth focused on the diameter increasing, which mainly involved in secondary growth. This result was consistent with the changes of histological chemistry in different internodes of stem.

### Illumina sequencing and alignment to the reference genome

The RNA-Seq was used to analyze differences in gene expression during different internodes. 15 cDNA libraries were constructed, IN3, IN5, IN7, IN9, IN11 with triple replication represented IN3-4, IN5-6, IN7-8, IN9-10, IN11-12 were adopted for RNA-seq and differential expression genes (DEGs) analysis. Each sample generated more than 16 million reads (Table 1), which was sufficient for the next quantitative analysis of gene expression. Then, gained reads were aligned to the *E. grandis* reference genome using SOAPaligner/soap2 software. Of the all internodes, over 85% reads could match with the reference genome. It was found that IN3 showed the least matched (87.78%) with 81.85% unique and 5.91% multiple genomic locations. However, there is still 12.22% were unmatched (Table 1). In other samples, the sequence reads could match over 90%, in which a unique genomic location approximately matched 90%. And the proportion of covered genome at different internodes ranged from 61.23% to 62.28% (Table 1), which suggested the RNA-Seq was sufficient to identify DEGs.

**Table 1:** Summary of read numbers based on the RNA-Seq data from *Eucalyptus* internodes

	IN3	IN5	IN7	IN9	IN11
Total reads	18,602,753	23,988,926	19,799,641	16,562,096	17,972,516
Mapped reads	16,329,497	22,228,139	18,706,701	15,671,055	17,081,079
	(87.78%)	(92.66%)	(94.48%)	(94.62%)	(95.04%)
Unique matched reads	15,592,203	19,970,855	17,706,827	14,859,830	16,120,472
	(81.85%)	(83.25%)	(89.43%)	(89.72%)	(89.70%)
Multi-position matched reads	1,125,528	2,196,171	994,932	807,336	953,109
	(5.91%)	(9.15%)	(5.03%)	(4.87%)	(5.30%)
Unmapped reads	2,273,256	1,760,787	1,092,940	891,041	891,437
	(12.22%)	(7.34%)	(5.52%)	(5.38%)	(4.96%)
Mapping gene number	22,621	22,638	22,612	22,490	22,258
Mapping-gene percent (%)	61.23%	62.23%	62.28%	62.21%	61.87%

### Annotation of all detected genes expressed during different stem internode

To facilitate the global analysis of gene expression, all predicted genes were assigned to different functional categories using agriGO (version 2.0) (http://systemsbiology.cau.edu.cn/agriGOv2/). The annotations were verified manually and integrated using gene ontology (GO) classification (http://www.geneontology.org).

**Fig. 2:**
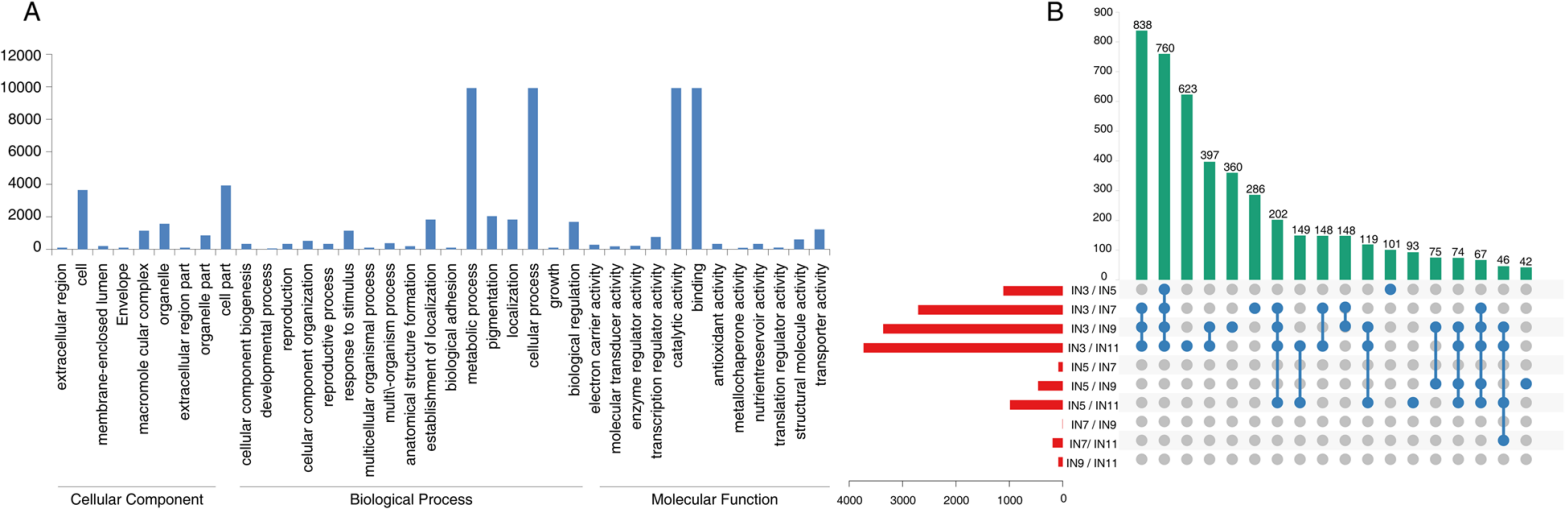
Expression profiling of DEGs during stem different development stages. **A** Venn diagrams representing the numbers of DEGs and the overlaps of sets obtained across three comparisons from two groups. **B** GO classification of Eucalyptus internode genes

Of 23,658 detected genes, 12,841 genes were categorized into 38 functional groups based on sequence homology. In each of the three main categories (cellular component, biological process and molecular function) of the GO classification, there were 9, 17 and 12 functional groups, respectively. Cell (GO:0005623) with 3,845 genes, were dominant in the main category of cellular component. Metabolic process (GO: 0008152), with 9,913 genes, were dominant in the main category of biological process. Binding (GO: 0005488) consisted of 11,357 genes, were dominant in the main categories of molecular function. We also noticed a high percentage of genes from functional groups of and cell part (GO: 0044464) with 3,845 genes, cellular process (GO: 0009987) with 7,718 genes, and catalytic activity (GO:0003824) consisted of 9,847 genes in the three main categories, respectively (Fig. 2A).

### Analysis of differentially expressed genes (DEGs) in different development stages

The DEGs of 5 internode samples (IN3, IN5, IN7, IN9, IN11) represented different developmental stage were tested in order to study the changes of from primary growth to secondary growth of stem in Eucalyptus. A total of 5,149 DEGs were obtained by pairwise comparison and the details listed in Supplemental Table 1. Compared with IN3, more and more DEGs were obtained with increasing secondary growth from IN5 to IN11 (Fig. 2B, Supplemental Table 1), which was corresponding with the change from primary to secondary growth of stem. Meanwhile, the least DEGs between IN7 and IN9 expressed slight changes of secondary growth. Meanwhile, overlapping sets of DEGs in these pairwise comparisons were also performed and 988 overlapping genes between IN3/IN7 and IN3/IN11, which also suggested the stem primarily underwent secondary growth from IN7. Only fewer DEGs (66) were overlapped between IN3/IN5 and IN3/IN7, which further verify the result of histological chemistry of stem (Fig. 1A). IN3 mainly focused on primary growth, IN5 underwent key transition from primary to secondary growth, and from IN7, the stem came into the secondary growth stage.

**Fig. 3:**
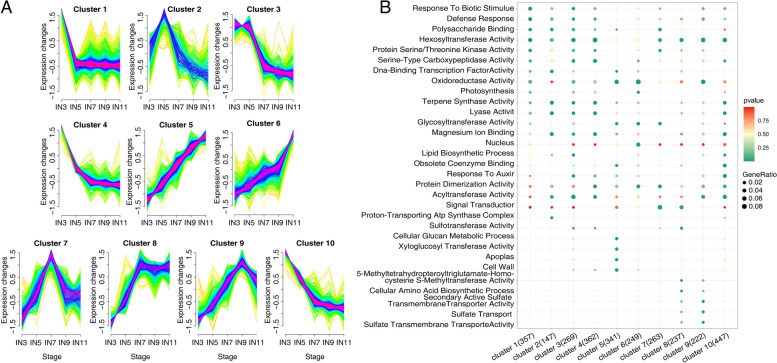
Mfuzz clustering and enrichment analysis of transcripts differentially expressed between the IN3-11. **A** Results of Mfuzz clustering of 5,149 differentially expressed genes (DEGs). **B** The top 30 Gene Ontology (GO) enrichment of selected category of differentially expressed genes (DEGs) based on Mfuzz cluster analysis. The larger dot indicates more genes. And the deeper red color indicates the smaller Q-value while the deeper green color indicates the higher Q-value

Mfuzz clustering analysis was carried out to investigate the DEGs changes from primary growth to secondary growth. All 5149 DEGs were grouped into 10 clusters. Clusters 5, 6 showed a similar tendency with increased gradually from the apex to IN5, and the expression level was highest in IN9-11, which indicates that these transcripts are potentially involved in secondary growth. Contrarily, expression trends of genes in cluster 4 and 10 declined gradually from the IN3 to IN9 and reached a steady state in IN11, which suggested these genes mainly involved in primary growth. Similarly, the clusters 1 decreased from the IN3 to IN5 and reached a steady state in IN5-11, which also potentially involved in primary growth (Fig. 3A, Supplemental Table 2). GO enrichment analysis was also carried out. Genes related to the cell wall, response to Auxin, and the DNA binding transcription factor activity were significantly enriched in cluster 5, cluster 10, and cluster 6, associated with the secondary growth. While transcripts that were predominantly expressed in the IN3 (cluster 1) potentially involved in primary growth function in pathways with DNA binding transcription factor activity, polysaccharide binding, hexosyltransferase activity and cytoskeleton organization (Fig. 3B).

Moreover, a total of 20 DEGs were selected for quantitative real-time PCR (The primers listed in Supplemental Table 3) to verify their expression levels. The result showed that the expression trends of these DEGs were similar with the results of RNA-Seq (Supplemental Fig. 1).

### Expression analysis of differentially expressed genes in lignin synthesis pathway

**Fig. 4:**
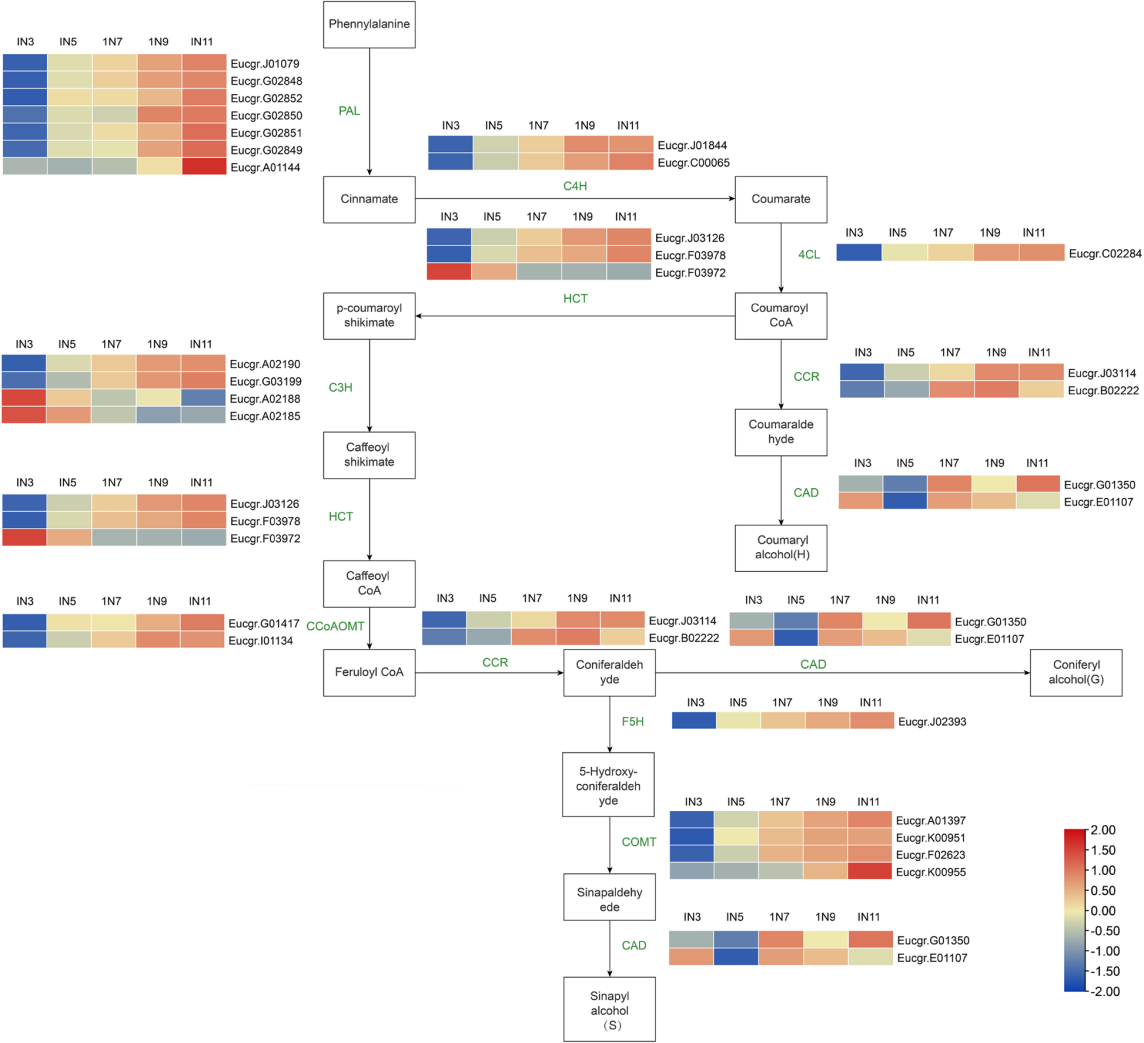
Lignin synthesis pathway map. The color scale represents the module-trait correlations from –1 (blue) to 1 (red). The color bar indicates expression and correlation levels from low(blue) to high (red). Panels IN3, IN5, IN7, IN9 and IN11 represent the different internodes stage, respectively. The necessary copyright authorization was secured to use the KEGG image shown in the figure

Lignin biosynthesis genes showed differential expression, which played essential roles in secondary growth in woody plants [2]. In this study, genes involving lignin biosynthesis also mapped and analyzed from primary to secondary growth (Supplemental Table 4). As shown in Fig. 4, 28 lignin biosynthesis key genes shown differential expression were identified. Among them, most of genes (25 genes) showed high level expression in IN7, IN9 and IN11 or gradual increases from IN3 to IN11 such as *EgrPAL*, *EgrC4H*, *EgrCOMT*, *EgrCAD*, and *EgrCCoAOMT*. These genes were mainly grouped into cluster 5 and cluster 6 (Fig. 3A), which showed gradual increases from IN3 to IN11. It also means that the expression level of the transcripts was corresponding with the gradual increases from primary to secondary growth. However, there are still several genes shown decreased from primary to secondary growth, such as *EgrC3H2* (Eucgr. A02188), *EgrC3H1* (Eucgr. A02185), *EgrHCT1* (Eucgr. F03972), suggesting that these genes might be associated with primary growth of stem in Eucalyptus and needs to be further verified.

### Weighted gene co-expression network analysis (WGCNA) identified hub genes involved in transition from primary to secondary growth

**Fig. 5:**
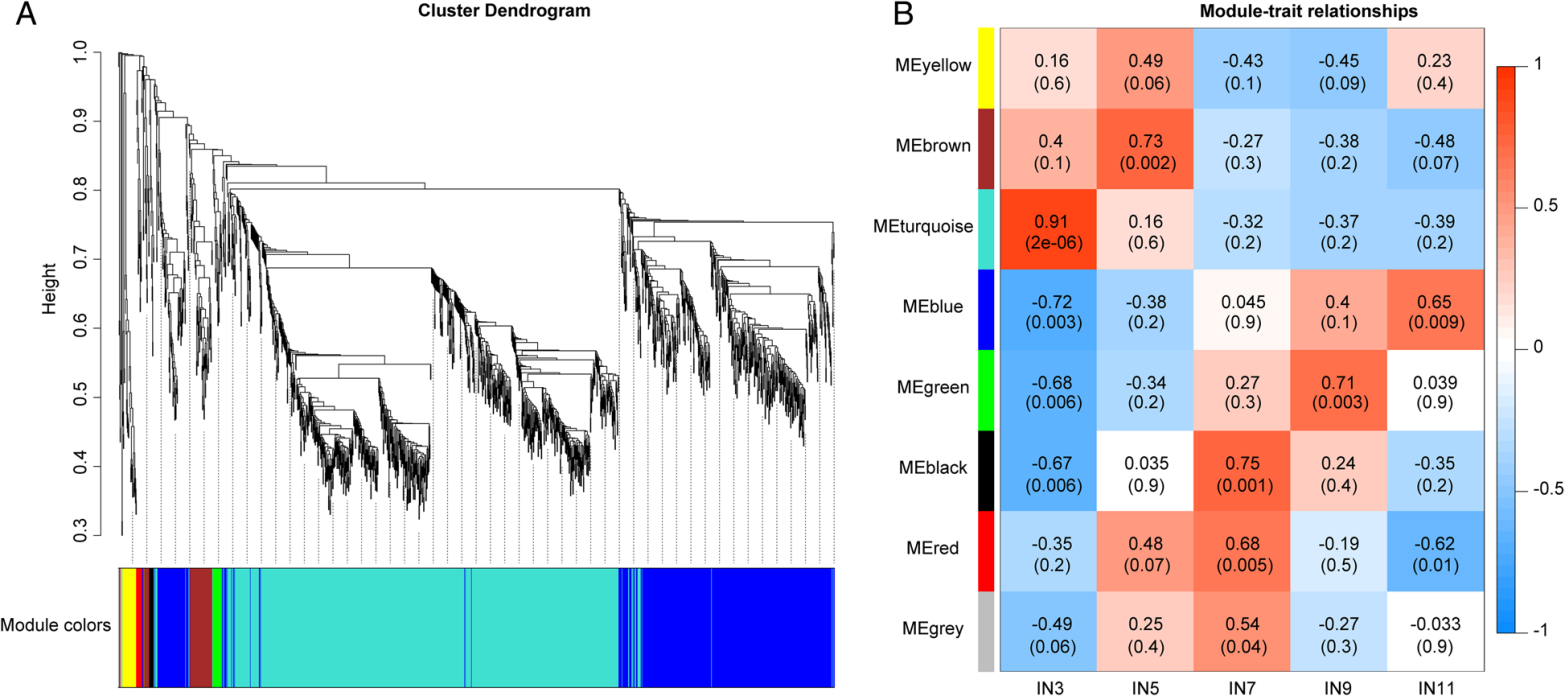
Weighted Gene Co-expression Network Analysis (WGCNA) of DEGs. **A** Cluster dendrogram of transcription factors based on expression levels in the five developmental stages (IN3, IN5, IN7, IN9 and IN11). Each branch represents a gene and each colour below represents a gene co-expression module. **B** Module-trait relationship analysis by WGCNA. Numbers in the squares presented the module-trait correlations (with corresponding *p*-values in parentheses). The color scale represents the module-trait correlations from –1 (blue) to 1 (red). The colour bar indicates expression and correlation levels from low(blue) to high (red). Panels IN3, IN5, IN7, IN9 and IN11 represent the different internodes stage, respectively

WGCNA was performed to get more understanding of the genes involving in the transition from primary to secondary growth, which would define clusters and obtain gene modules with specific patterns of expression during stem development stages. After removal of the genes with low fluctuation in expression (standard deviation ≤ 0.2), 1,545 of 5,149 DEGs were subjected to pairwise correlation analysis regarding gene expression and sorted into different modules, highly correlated gene clusters. The genes in the same modules shared high correlation coefficients, constructing eight modules based on expression pattern and specificity in Eucalyptus (Fig. 5; Supplemental Table 5). The turquoise module was highly positively correlated with all the stages of stem development involved with primary growth (*r*^2^ = 0.92 and *p* value < 0.001), which 868 DEGs belong to the turquoise module and their peak expression in IN3 (Fig. 5 and Supplemental Table 5). It was also found that several key transcription factors, such as MYB, YAB2, AP1 and HB family [28–31], which involved in regulating primary growth and some key hemicellulose and lignin synthesis enzymes including UDP, UGT, CCOAMT, LAC, and EXP were also identified [32].

**Fig. 6:**
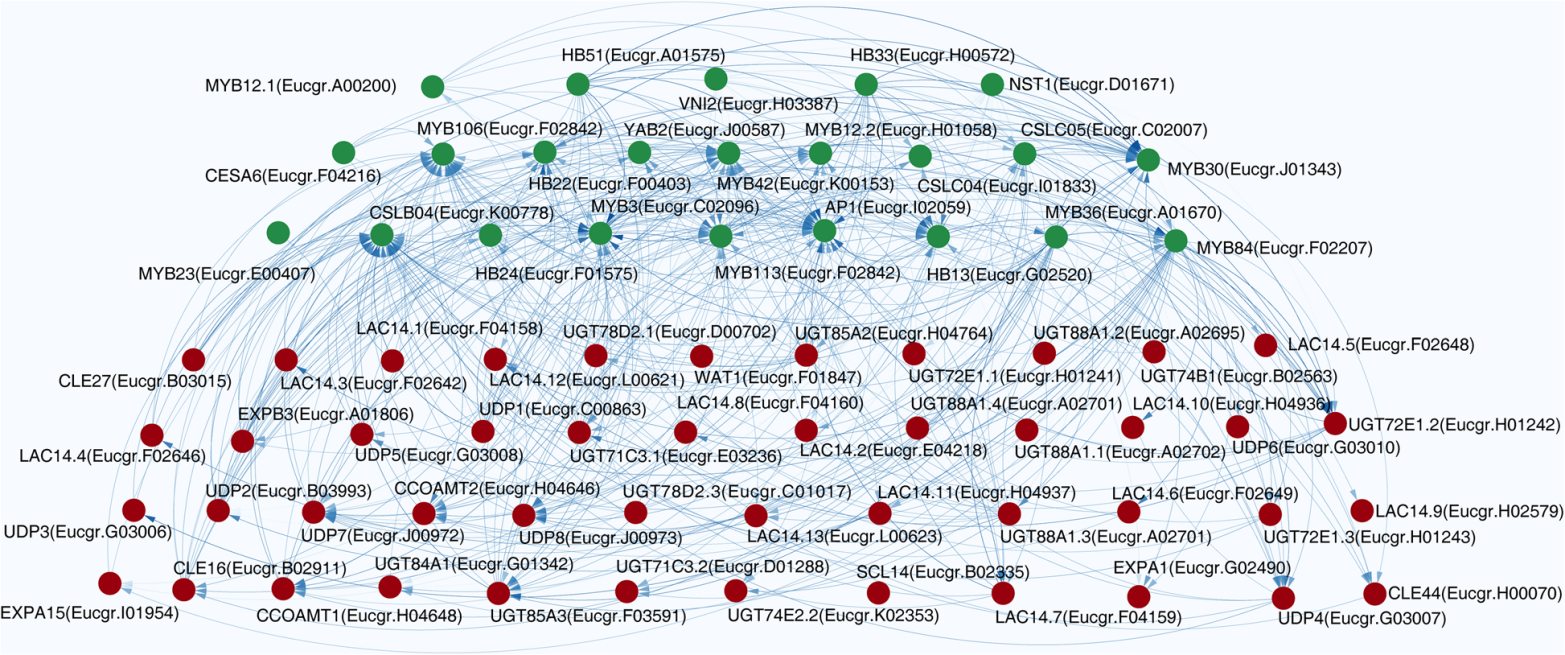
Co-expression network analysis of 70 selected genes from the hub genes in turquoise module. Green dots represent TFs, while red dots represent genes. The lines represent the connected weights between genes, and the depth represents the weight value. And the arrow indicates the direction of control

To further select the hub genes involving the secondary growth in Eucalyptus, the genes with the weight value greater than 0.5 were selected to perform. About 70 genes were screened out and their expression trend was detailed in Supplemental Fig. 2 and Supplemental Table 6 and the network diagram of the screened genes was drawn (Fig. 6 and Supplemental Table 7). The transcription factors NST1, HB51, VNI2, MYB12.1 and HB33 are located in the first layer, which formed a hierarchy control to regulate downstream genes related to lignin and cellulose synthesis (such as *EgrUDP, EgrUGT, EgrLAC, EgrCCOAMT)* and finally regulated secondary growth in Eucalyptus (Supplemental Fig. 3 and Supplemental Table 8).

## Discussion

Eucalyptus, as a hardwood species, was widely cultivated in worldwide and had the superior character of fast growth and high quality of wood. Several genes involved in secondary growth including cell wall formation, cellulose and lignin synthesis were discovered [33–36]. For example, EgMYB1 and EgMYB2 could bind specifically to the promoter region of lignin synthesis gene such as Coenzyme A Reductase (CCR) and cinnamyl alcohol dehydrogenase (CAD) and further regulate secondary wall biosynthesis [12, 37, 38]. In addition, EcHB1 of the HD-ZIP Class II family TFs and LIM transcription factor were also found to enhance xylem cell wall biosynthesis [39, 40]. However, there are still a large number of valuable genes with potential breeding need to be discovered. In this study, a unique system of internodes from apex to bottom of stem in Eucalyptus which represent from primary to secondary growth combining transcriptome sequencing were adopted as identifying important genes involved in secondary growth. Based on these, selected genes involved secondary growth were originated dynamic changes progress, which was different with previously selected genes which expressed in specific tissues such as xylem or tension wood [1, 6–9]. This provided more effective approach to explore wood formation-associated gene and more easily understand secondary growth or wood formation developmental processes in Eucalyptus.

WGCNA could find clusters (modules) of highly correlated genes to external sample traits, which widely used in RNA-seq analysis [22, 41]. In the present study, 868 genes clustered a module, which correlated with secondary growth were identified by the correlation algorithm. Among these genes, some key TFs and genes were also observed. For example, Transcription factors include R2R3-MYB, EgMYB1, SND2, EgLBDs. CBFs, EgrNAC26, EgrNAC61, EgNAC141 were identified, which showed highly correlated with secondary growth in Eucalyptus [14, 34, 37, 42–44]. It was confirmed that MYB family acted a master role in wood formation by activating the biosynthesis of cellulose, lignin or hemicellulose [45–50]. As we predicted, EgMYB1 and EgMYB2 also regulated secondary cell wall formation in Eucalyptus [37, 38], whose homology genes were also identified in this study. Plant secondary growth is regulated by transcriptional networks composed of transcription factors and secondary cell wall synthetase genes [35, 39]. MYB3 and MYB42 directly activate lignin biosynthesis genes during secondary wall formation in poplar [10] and Arabidopsis [51]. Several studies have revealed the transcriptional regulatory network mediated by NAC family transcription factors NST (SND) and VND in Arabidopsis wood formation [52–54]. In this study, NST1 and VNI2 which belong to the NAC family and can regulate the process of secondary wall thickening and ductal cell formation of fibrocyte [55–59] showed increasing from primary to secondary growth.

In previous study, YAB2 and AP1 are related to apical meristem development while HB family transcription factors are mainly involved in the regulation of apical meristem and vascular tissue formation [28, 29]. YABBY is also involved in the signaling between the blades and SAM, which located on the margins of SAM and are distinguished from the *WUS* and *CLV3*-labeled central meristem [60, 61]. Differently, YAB2 expressed in the young leaf primordium, which can regulate the polarity development of lateral branches and stem apex meristem development [62–64]. As for AP1, the deletion of *GmAP1* increased the number of main stem segments and plant height while overexpressing *GmAP1a* showed decreased number of main stem segments, decreased plant height, which indicated that *GmAP1* was involved in the regulation of soybean plant height growth [30]. Moreover, HB genes are associated with shoot apex meristem development [65] and secondary growth [29]. In the end, AP1, YAB2, EXP were also identified as hub genes involving in primary growth in our study, which further verify our accurate result.

Here, some cluster2 genes played an important role in the transition from primary growth to secondary growth through the temporal analysis. *LRR*, *UGT*, *LAC*, *DUF247* and *TCP11* genes were found to be closely related to the transition from primary growth to secondary growth. Among them, *LAC* and *UGT* genes were closely related to lignin formation [32, 66, 67]. Interestingly, *AtDUF247* was expressed particularly in vascular and developing tissues, which was necessary to maintain normal cell wall composition and structure by affecting the expression of other gene members in Arabidopsis [68]. Clearly, a number of LRR-RLKs genes were required in secondary vascular development [69]. As a member of LRR-RLK, PXC1 was involved in secondary cell wall formation in xylem fibers, which showed similar function in shoot apical meristem [70]. Besides, TCP11 was also verified as involving in the growth and development of vascular bundles [71].

The processes of secondary cell wall formation mainly include the biosynthesis and assembly of four components: polysaccharides (mainly cellulose and hemicellulose), lignin, cell wall proteins, and some secondary substances, such as flavonoids, tannic acid, pectin, etc. [3, 4]. During the secondary growth transition, the contents and structure lignin, cellulose and hemicellulose also was going to be the different. Such as lignin and cellulose contents will increase. Correspondingly, CCOAMT and LAC which were involved in the regulation of lignin synthesis were identified and showed highly enrichment in secondary growth. CesA1, CesA3 and CesA6 are mainly responsible for the synthesis of cellulose in the primary wall [3, 72], while CesA4, CesA7 and CesA8 are mainly responsible for the synthesis of cellulose in the secondary wall [73–75]. CLE and SCL are associated with cellulose synthesis [76, 77], CESA6 which closely related to primary wall also mainly expressed in primary growth stage [78, 79]. UDP and UGT are related to hemicellulose synthesis were also explore [66, 67]. While *PttEXP1* gene was more active in the secondary growth of mature stem segments [80, 81]. These results were consistent with the selected genes in this study that may be responsible for the transition from primary to secondary growth.

What’s more, transcription factors PtrMYB021 and PtrMYB002 located in the second layer of regulatory network could directly regulate the expression of a series of genes related to the synthesis of cell wall components downstream such as PtrLAC14, PtrLAC15, PtrLAC40, PtrLAC41, PtrLAC49 [82]. Similar with the poplar, key MYB transcript factor as upstream and several *LAC* genes as downstream were also found in Eucalyptus and the hierarchy relationship also showed similar, which suggested similar regulating mechanism existed in woody plants. Hence, these results laid a basis for elucidating the molecular mechanism of secondary growth in Eucalyptus and these selected genes are a potential resource to further molecular breeding in Eucalyptus.

## Conclusions

In present research, RNA-Seq was adopted to profile changes in gene expression from the apex to bottom of stem which represented from primary to secondary growth in Eucalyptus. The typical developmental stages were harvested to analyze the changing of cell structure and gene expression to systematically elucidate the transition from primary to secondary growth in Eucalyptus. 5,149 DEGs were identified, in which 70 hub genes were further optimized with high connectivity closely related to primary or secondary growth. These genes involved in Cell wall, response to auxin and DNA binding transcription factor activity. Transcription factors AP1, YAB2 and *EXP* genes which involved in primary growth mainly expressed in the IN3 while NAC, MYB, CESA6 highly affected in secondary growth were enriched in IN9 to 11 which mainly underwent secondary growth. These genes expressed predominantly in primary and secondary growth zone which have different regulatory cascades, and further formed a hierarchy regulatory network to regulate wood formation in Eucalyptus. These provide a potential resource to study secondary growth, especially the key transition from primary to secondary growth. Furthermore, exploration of these key genes involved in secondary growth will be great useful to future molecular breeding in Eucalyptus.

### Supplementary Information

**Additional file 1: Supplemental Table 1.** The expression of DEGs (TPM) in different internodes. **Supplemental Table 2.** The result-cluster data of Mfuzz analysis. **Supplemental Table 3.** The primers of selected genes used for qRT-PCR. **Supplemental Table 4.** Gene names and accession numbers in lignin biosynthesis. **Supplemental Table 5.** The result data of WGCNA analysis. **Supplemental Table 6.** 70 selected genes with annotation. **Supplemental Table 7. **Gene names and accession numbers in the network map. **Supplemental Table 8.** Gene names and accession numbers in cellulose and xylan biosynthesis.

**Additional file 2: Supplemental Figure 1.** Validation of relative expression level of 20 selected DEGs by RT-qPCR. Values are means ± SD of three biological replicates. Panels IN3, IN5, IN7, IN9 and IN11 represent the different internodes stage, respectively. Error bar represents standard deviation (*n*=3).

**Additional file 3: Supplemental Figure 2.** The heat-map of turquoise module genes and the heat map of 70 selected genes from the hub genes in turquoise module. The color bar indicates expression and correlation levels from low(blue) to high (red). Panels IN3, IN5, IN7, IN9 and IN11 represent the different internodes stage, respectively.

**Additional file 4: Supplemental Figure 3.** Cellulose synthesis pathway map. The color bar indicates expression and correlation levels from low(blue) to high (red). Panels IN3, IN5, IN7, IN9 and IN11 represent the different internodes stage, respectively. The necessary copyright authorization was secured to use the KEGG image shown in the figure.

## Data Availability

The datasets generated and/or analyzed during the current study are available in the NCBI SRA repository, with accession number PRJNA1012557 (https://www.ncbi.nlm.nih.gov/bioproject/PRJNA1012557). All data generated or analyzed during this study are included in this published article and its supplementary information files.

## References

[1] Paiva JA, Prat E, Vautrin S, Santos MD, San-Clemente H, Brommonschenkel S, Fonseca PG, Grattapaglia D, Song X, Ammiraju JS (2011). Advancing Eucalyptus genomics: identification and sequencing of lignin biosynthesis genes from deep-coverage BAC libraries. BMC Genomics.

[2] Myburg AA, Grattapaglia D, Tuskan GA, Hellsten U, Hayes RD, Grimwood J, Jenkins J, Lindquist E, Tice H, Bauer D (2014). The genome of Eucalyptus grandis. Nature.

[3] Doblin MS, Kurek I, Jacob-Wilk D, Delmer DP (2002). Cellulose biosynthesis in plants: from genes to rosettes. Plant Cell Physiol.

[4] Kalluri UC, Joshi CP (2003). Isolation and characterization of a new, full-length cellulose synthase cDNA, PtrCesA5 from developing xylem of aspen trees. J Exp Bot.

[5] Paux E, Carocha V, Marques C (2005). Mendes de Sousa A, Borralho N, Sivadon P, Grima-Pettenati J: Transcript profiling of Eucalyptus xylem genes during tension wood formation. New Phytol.

[6] Foucart C, Paux E, Ladouce N, San-Clemente H, Grima-Pettenati J, Sivadon P (2006). Transcript profiling of a xylem vs phloem cDNA subtractive library identifies new genes expressed during xylogenesis in Eucalyptus. New Phytol.

[7] Rengel D, Clemente HS, Servant F, Ladouce N, Paux E, Wincker P, Couloux A, Sivadon P, Grima-Pettenati J (2009). A new genomic resource dedicated to wood formation in Eucalyptus. BMC Plant Biol.

[8] Mizrachi E, Hefer CA, Ranik M, Joubert F, Myburg AA (2010). De novo assembled expressed gene catalog of a fast-growing Eucalyptus tree produced by Illumina mRNA-Seq. BMC Genomics.

[9] Hefer CA, Mizrachi E, Myburg AA, Douglas CJ, Mansfield SD (2015). Comparative interrogation of the developing xylem transcriptomes of two wood-forming species: P opulus trichocarpa and E ucalyptus grandis. New Phytol.

[10] McCarthy RL, Zhong R, Fowler S, Lyskowski D, Piyasena H, Carleton K, Spicer C, Ye Z-H (2010). The poplar MYB transcription factors, PtrMYB3 and PtrMYB20, are involved in the regulation of secondary wall biosynthesis. Plant Cell Physiol.

[11] Spokevicius AV, Southerton SG, MacMillan CP, Qiu D, Gan S, Tibbits JF, Moran GF, Bossinger G (2007). β-tubulin affects cellulose microfibril orientation in plant secondary fibre cell walls. Plant J.

[12] Legay S, Lacombe E, Goicoechea M, Briere C, Séguin A, Mackay J, Grima-Pettenati J (2007). Molecular characterization of EgMYB1, a putative transcriptional repressor of the lignin biosynthetic pathway. Plant Sci.

[13] Lopes FJF, Pauly M, Brommonshenkel SH, Lau EY, Diola V, Passos JL, Loureiro ME (2010). The EgMUR3 xyloglucan galactosyltransferase from Eucalyptus grandis complements the mur3 cell wall phenotype in Arabidopsis thaliana. Tree Genet Genomes.

[14] Soler M, Plasencia A, Larbat R, Pouzet C, Jauneau A, Rivas S, Pesquet E, Lapierre C, Truchet I, Grima-Pettenati J (2017). The Eucalyptus linker histone variant EgH1. 3 cooperates with the transcription factor EgMYB1 to control lignin biosynthesis during wood formation. New Phytologist.

[15] Liu B, Liu J, Yu J, Wang Z, Sun Y, Li S (2021). Lin Y-CJ, Chiang VL, Li W, Wang JP: Transcriptional reprogramming of xylem cell wall biosynthesis in tension wood. Plant Physiol.

[16] Dai X, Zhai R, Lin J, Wang Z, Meng D, Li M, Mao Y, Gao B, Ma H, Zhang B (2023). Cell-type-specific PtrWOX4a and PtrVCS2 form a regulatory nexus with a histone modification system for stem cambium development in Populus trichocarpa. Nature Plants.

[17] Ko J-H, Han K-H, Park S, Yang J (2004). Plant body weight-induced secondary growth in Arabidopsis and its transcription phenotype revealed by whole-transcriptome profiling. Plant Physiol.

[18] Prassinos C, Ko J-H, Yang J, Han K-H (2005). Transcriptome profiling of vertical stem segments provides insights into the genetic regulation of secondary growth in hybrid aspen trees. Plant Cell Physiol.

[19] Dharmawardhana P, Brunner AM, Strauss SH (2010). Genome-wide transcriptome analysis of the transition from primary to secondary stem development in Populus trichocarpa. BMC Genomics.

[20] Sundell D, Street NR, Kumar M, Mellerowicz EJ, Kucukoglu M, Johnsson C, Kumar V, Mannapperuma C, Delhomme N, Nilsson O (2017). AspWood: high-spatial-resolution transcriptome profiles reveal uncharacterized modularity of wood formation in Populus tremula. Plant Cell.

[21] Chao Q, Gao ZF, Zhang D, Zhao BG, Dong FQ, Fu CX, Liu LJ, Wang BC (2019). The developmental dynamics of the Populus stem transcriptome. Plant Biotechnol J.

[22] Langfelder P, Horvath S (2008). WGCNA: an R package for weighted correlation network analysis. BMC Bioinformatics.

[23] Love MI, Huber W, Anders S (2014). Moderated estimation of fold change and dispersion for RNA-seq data with DESeq2. Genome Biol.

[24] Chen C, Chen H, Zhang Y, Thomas HR, Frank MH, He Y, Xia R (2020). TBtools: An Integrative Toolkit Developed for Interactive Analyses of Big Biological Data. Mol Plant.

[25] Kumar L (2007). M EF: Mfuzz: a software package for soft clustering of microarray data. Bioinformation.

[26] de Oliveira LA, Breton MC, Bastolla FM (2012). Camargo SdS, Margis R, Frazzon J, Pasquali G: Reference genes for the normalization of gene expression in Eucalyptus species. Plant Cell Physiol.

[27] Livak  KJ, Schmittgen TD (2001). Analysis of relative gene expression data using real-time quantitative PCR and the 2− ΔΔCT method. Methods.

[28] Robischon M, Du J, Miura E, Groover A (2011). The Populus class III HD ZIP, popREVOLUTA, influences cambium initiation and patterning of woody stems. Plant Physiol.

[29] Zhu Y, Song D, Sun J, Wang X, Li L (2013). PtrHB7, a class III HD-Zip gene, plays a critical role in regulation of vascular cambium differentiation in Populus. Mol Plant.

[30] Chen L, Nan H, Kong L, Yue L, Yang H, Zhao Q, Fang C, Li H, Cheng Q, Lu S (2020). Soybean AP1 homologs control flowering time and plant height. J Integr Plant Biol.

[31] van der Graaff E, Laux T, Rensing SA (2009). The WUS homeobox-containing (WOX) protein family. Genome Biol.

[32] Nakano Y, Yamaguchi M, Endo H, Rejab NA, Ohtani M (2015). NAC-MYB-based transcriptional regulation of secondary cell wall biosynthesis in land plants. Front Plant Sci.

[33] Feltrim D, Gupta B, Gundimeda S, Kiyota E, Júnior APD, Cintra LC, Mazzafera P (2022). Exposure of Eucalyptus to varied temperature and CO2 has a profound effect on the physiology and expression of genes related to cell wall formation and remodeling. Tree Genet Genomes.

[34] Lu Q, Shao F, Macmillan C, Wilson IW, Van der Merwe K, Hussey SG, Myburg AA, Dong X, Qiu D (2018). Genomewide analysis of the lateral organ boundaries domain gene family in Eucalyptus grandis reveals members that differentially impact secondary growth. Plant Biotechnol J.

[35] Chen H, Wang JP, Liu H, Li H (2019). Lin Y-CJ, Shi R, Yang C, Gao J, Zhou C, Li Q: Hierarchical transcription factor and chromatin binding network for wood formation in Populus trichocarpa. Plant Cell.

[36] Han X, Zhao Y, Chen Y, Xu J, Jiang C, Wang X, et al. Lignin biosynthesis and accumulation in response to abiotic stresses in woody plants. Forestry Res. 2022;2:9. 10.48130/FR-2022-0009 PMC11524291 39525415

[37] Legay S, Sivadon P, Blervacq AS, Pavy N, Baghdady A, Tremblay L, Levasseur C, Ladouce N, Lapierre C, Séguin A (2010). EgMYB1, an R2R3 MYB transcription factor from eucalyptus negatively regulates secondary cell wall formation in Arabidopsis and poplar. New Phytol.

[38] Goicoechea M, Lacombe E, Legay S, Mihaljevic S, Rech P, Jauneau A, Lapierre C, Pollet B, Verhaegen D, Chaubet-Gigot N (2005). EgMYB2, a new transcriptional activator from Eucalyptus xylem, regulates secondary cell wall formation and lignin biosynthesis. Plant J.

[39] Luo L, Li L. Molecular understanding of wood formation in trees. Forestry Res. 2022;2:5. 10.48130/FR-2022-0005 PMC11524228 39525426

[40] Li Y, Yang Z, Zhang Y, Guo J, Liu L, Wang C, Wang B, Han G (2022). The roles of HD-ZIP proteins in plant abiotic stress tolerance. Front Plant Sci.

[41] Toubiana D, Puzis R, Sadka A, Blumwald E (2019). A genetic algorithm to optimize weighted gene co-expression network analysis. J Comput Biol.

[42] Hussey SG, Mizrachi E, Spokevicius AV, Bossinger G, Berger DK, Myburg AA (2011). SND2, a NAC transcription factor gene, regulates genes involved in secondary cell wall development in Arabidopsis fibres and increases fibre cell area in Eucalyptus. BMC Plant Biol.

[43] Laubscher M, Brown K, Tonfack L, Myburg AA, Mizrachi E, Hussey S (2018). Temporal analysis of Arabidopsis genes activated by Eucalyptus grandis NAC transcription factors associated with xylem fibre and vessel development. Sci Rep.

[44] Sun Y, Jiang C, Jiang R, Wang F, Zhang Z, Zeng J (2021). A novel NAC transcription factor from Eucalyptus, EgNAC141, positively regulates lignin biosynthesis and increases lignin deposition. Front Plant Sci.

[45] Yoshida K, Ma D, Constabel CP (2015). The MYB182 protein down-regulates proanthocyanidin and anthocyanin biosynthesis in poplar by repressing both structural and regulatory flavonoid genes. Plant Physiol.

[46] Tang X, Zhuang Y, Qi G, Wang D, Liu H, Wang K, Chai G, Zhou G (2015). Poplar PdMYB221 is involved in the direct and indirect regulation of secondary wall biosynthesis during wood formation. Sci Rep.

[47] Ma D, Reichelt M, Yoshida K, Gershenzon J, Constabel CP (2018). Two R2R3-MYB proteins are broad repressors of flavonoid and phenylpropanoid metabolism in poplar. Plant J.

[48] Yang L, Zhao X, Ran L, Li C, Fan D, Luo K (2017). PtoMYB156 is involved in negative regulation of phenylpropanoid metabolism and secondary cell wall biosynthesis during wood formation in poplar. Sci Rep.

[49] Gui J, Luo L, Zhong Y, Sun J, Umezawa T, Li L (2019). Phosphorylation of LTF1, an MYB transcription factor in Populus, acts as a sensory switch regulating lignin biosynthesis in wood cells. Mol Plant.

[50] Jiao B, Zhao X, Lu W, Guo L, Luo K (2019). The R2R3 MYB transcription factor MYB189 negatively regulates secondary cell wall biosynthesis in Populus. Tree Physiol.

[51] Geng P, Zhang S, Liu J, Zhao C, Wu J, Cao Y, Fu C, Han X, He H, Zhao Q (2019). MYB20, MYB42, MYB43, and MYB85 Regulate Phenylalanine and Lignin Biosynthesis during Secondary Cell Wall Formation1 [OPEN]. Plant Physiol.

[52] Zhong R, Richardson EA, Ye Z-H (2007). The MYB46 transcription factor is a direct target of SND1 and regulates secondary wall biosynthesis in Arabidopsis. Plant Cell.

[53] Zhou J, Lee C, Zhong R, Ye Z-H (2009). MYB58 and MYB63 are transcriptional activators of the lignin biosynthetic pathway during secondary cell wall formation in Arabidopsis. Plant Cell.

[54] Li Z, Omranian N, Neumetzler L, Wang T, Herter T, Usadel B, Demura T, Giavalisco P, Nikoloski Z, Persson S (2016). A transcriptional and metabolic framework for secondary wall formation in Arabidopsis. Plant Physiol.

[55] McCarthy RL, Zhong R, Ye Z-H (2009). MYB83 is a direct target of SND1 and acts redundantly with MYB46 in the regulation of secondary cell wall biosynthesis in Arabidopsis. Plant Cell Physiol.

[56] Zhong R, Lee C, Ye Z-H (2010). Functional characterization of poplar wood-associated NAC domain transcription factors. Plant Physiol.

[57] Ohtani M, Nishikubo N, Xu B, Yamaguchi M, Mitsuda N, Goué N, Shi F, Ohme-Takagi M, Demura T (2011). A NAC domain protein family contributing to the regulation of wood formation in poplar. Plant J.

[58] Zhou J, Zhong R, Ye Z-H (2014). Arabidopsis NAC domain proteins, VND1 to VND5, are transcriptional regulators of secondary wall biosynthesis in vessels. PLoS ONE.

[59] Yan C, Nie Z, Hu Z, Huang H, Ma X, Li S, et al. Tissue-specific transcriptomics reveals a central role of CcNST1 in regulating the fruit lignification pattern in Camellia chekiangoleosa, a woody oil-crop. Forestry Res. 2022;2:10. 10.48130/FR-2022-0010 PMC11524261 39525417

[60] Eshed Y, Izhaki A, Baum SF, Floyd SK, Bowman JL (2004). Asymmetric leaf development and blade expansion in Arabidopsis are mediated by KANADI and YABBY activities. Development.

[61] Kumaran MK, Bowman JL, Sundaresan V (2002). YABBY polarity genes mediate the repression of KNOX homeobox genes in Arabidopsis. Plant Cell.

[62] Jie G (2022). Zhou X-t, Dai K-l, Yuan X-y, Guo P-y, Shi W-p, Zhou M-x: Comprehensive analysis of YABBY gene family in foxtail millet (Setaria italica) and functional characterization of SiDL. J Integr Agric.

[63] Liu X, Liao X-Y, Zheng Y, Zhu M-J, Yu X, Jiang Y-T, Zhang D-Y, Ma L, Xu X-Y, Liu Z-J (2021). Genome-Wide Identification of the YABBY Gene Family in Seven Species of Magnoliids and Expression Analysis in Litsea. Plants.

[64] Stahle MI, Kuehlich J, Staron L, von Arnim AG, Golz JF (2009). YABBYs and the transcriptional corepressors LEUNIG and LEUNIG_HOMOLOG maintain leaf polarity and meristem activity in Arabidopsis. Plant Cell.

[65] Long JA, Moan EI, Medford JI, Barton MK (1996). A member of the KNOTTED class of homeodomain proteins encoded by the STM gene of Arabidopsis. Nature.

[66] Su J, Zhang C, Zhu L, Yang N, Yang J, Ma B, Ma F, Li M (2021). MdFRK2-mediated sugar metabolism accelerates cellulose accumulation in apple and poplar. Biotechnol Biofuels.

[67] Ruprecht C, Blaukopf M, Pfrengle F (2022). Synthetic fragments of plant polysaccharides as tools for cell wall biology. Curr Opin Chem Biol.

[68] Wannitikul P, Wattana-Amorn P, Sathitnaitham S, Sakulkoo J, Suttangkakul A, Wonnapinij P, Bassel GW, Simister R, Gomez LD, Vuttipongchaikij S (2023). Disruption of a DUF247 Containing Protein Alters Cell Wall Polysaccharides and Reduces Growth in Arabidopsis. Plants.

[69] Hirakawa Y, Shinohara H, Kondo Y, Inoue A, Nakanomyo I, Ogawa M, Sawa S, Ohashi-Ito K, Matsubayashi Y, Fukuda H (2008). Non-cell-autonomous control of vascular stem cell fate by a CLE peptide/receptor system. Proc Natl Acad Sci.

[70] Wang J, Kucukoglu M, Zhang L, Chen P, Decker D, Nilsson O, Jones B, Sandberg G, Zheng B (2013). The Arabidopsis LRR-RLK, PXC1, is a regulator of secondary wall formation correlated with the TDIF-PXY/TDR-WOX4 signaling pathway. BMC Plant Biol.

[71] Huang T, Irish VF (2015). Temporal control of plant organ growth by TCP transcription factors. Curr Biol.

[72] Mazarei M, Baxter HL, Li M, Biswal AK, Kim K, Meng X, Pu Y, Wuddineh WA, Zhang J-Y, Turner GB, et al. Functional Analysis of Cellulose Synthase CesA4 and CesA6 Genes in Switchgrass (Panicum virgatum) by Overexpression and RNAi-Mediated Gene Silencing. Front Plant Sci. 2018;9:1114. 10.3389/fpls.2018.01114 PMC6088197 30127793

[73] Zhong R, Kandasamy MK, Ye Z-H (2021). XND1 Regulates Secondary Wall Deposition in Xylem Vessels through the Inhibition of VND Functions. Plant Cell Physiol.

[74] Nayeri S, Baghban Kohnehrouz B, Ahmadikhah A, Mahna N (2022). CRISPR/Cas9-mediated P-CR domain-specific engineering of CESA4 heterodimerization capacity alters cell wall architecture and improves saccharification efficiency in poplar. Plant Biotechnol J.

[75] Abbas M, Peszlen I, Shi R, Kim H, Katahira R, Kafle K, Xiang Z, Huang X, Min D, Mohamadamin M (2020). Involvement of CesA4, CesA7-A/B and CesA8-A/B in secondary wall formation in Populus trichocarpa wood. Tree Physiol.

[76] Willoughby AC, Nimchuk ZL (2021). WOX going on: CLE peptides in plant development. Curr Opin Plant Biol.

[77] Verbančič J, Lunn JE, Stitt M, Persson S (2018). Carbon Supply and the Regulation of Cell Wall Synthesis. Mol Plant.

[78] De Caroli M, Rampino P, Pecatelli G, Girelli CR, Fanizzi FP, Piro G, Lenucci MS. Expression of Exogenous GFP-CesA6 in Tobacco Enhances Cell Wall Biosynthesis and Biomass Production. In: Biology. vol. 11; 2022. 10.3390/biology11081139 PMC9405164 36009766

[79] O’Leary BM (2020). Another Brick in the Plant Cell Wall: Characterization of Arabidopsis CSLD3 Function in Cell Wall Synthesis[OPEN]. Plant Cell.

[80] Gray-Mitsumune M, Mellerowicz EJ, Abe H, Schrader J, Winzéll A, Sterky F, Blomqvist K, McQueen-Mason S, Teeri TT, Sundberg B (2004). Expansins abundant in secondary xylem belong to subgroup A of the alpha-expansin gene family. Plant Physiol.

[81] Cosgrove DJ (2000). New genes and new biological roles for expansins. Curr Opin Plant Biol.

[82] Lin Y-C, Li W, Sun Y-H, Kumari S, Wei H, Li Q, Tunlaya-Anukit S, Sederoff RR, Chiang VL (2013). SND1 transcription factor–directed quantitative functional hierarchical genetic regulatory network in wood formation in Populus trichocarpa. Plant Cell.

