# Supplementary figures and images for: Transcriptome analysis of the transition from primary to secondary growth of vertical stem in Eucalyptus grandis
Source: BMC Plant Biol. 2024 Feb 8;24:96. doi: 10.1186/s12870-024-04731-3 (PMC10851593; doi:10.1186/s12870-024-04731-3)

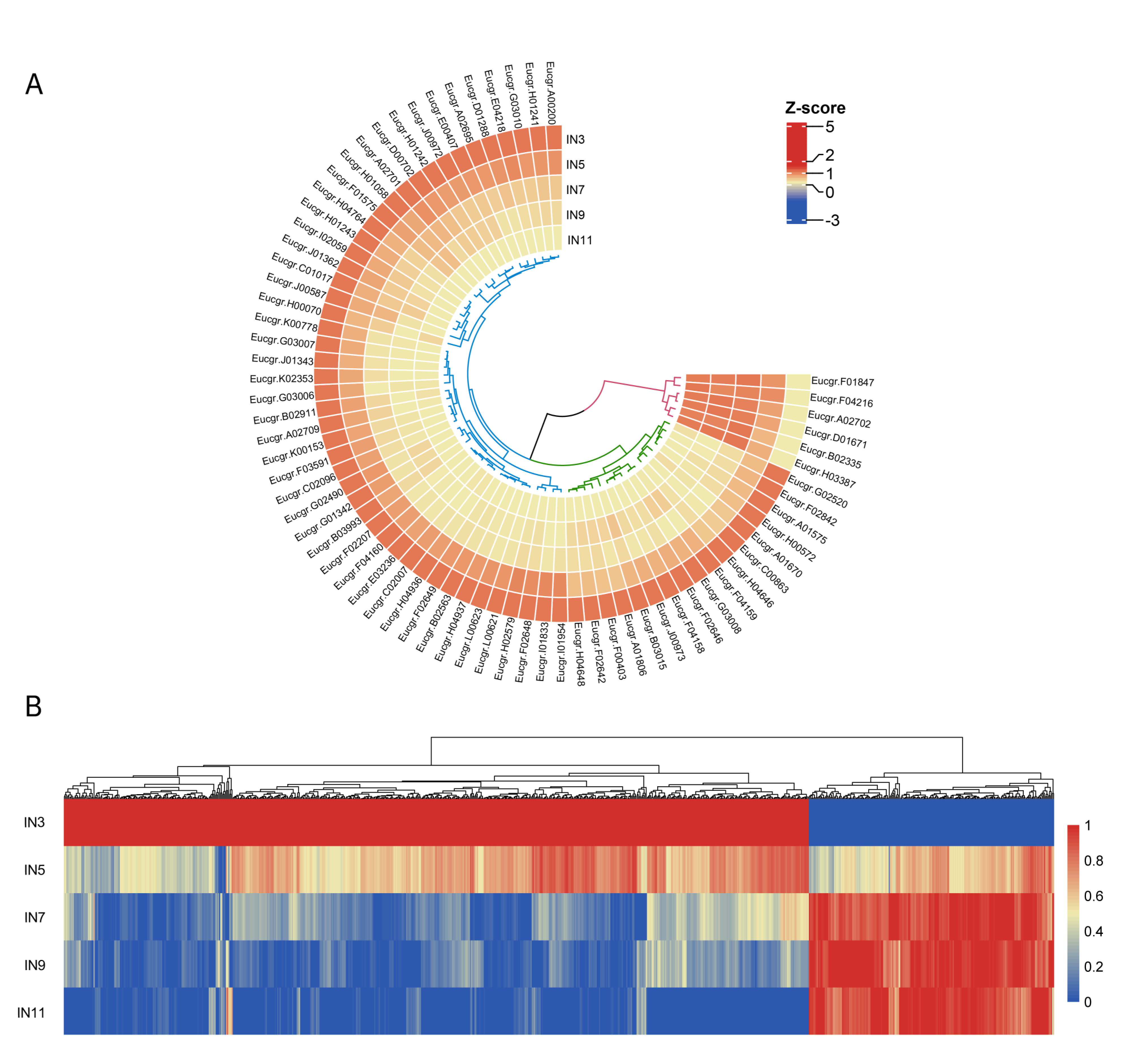

Supplement: Supplementary file 2 — Additional file 2: Supplemental Figure 1. Validation of relative expression level of 20 selected DEGs by RT-qPCR. Values are means ± SD of three biological replicates. Panels IN3, IN5, IN7, IN9 and IN11 represent the different internodes stage, respectively. Error bar represents standard deviation (n=3). [file 12870_2024_4731_MOESM2_ESM.tif]

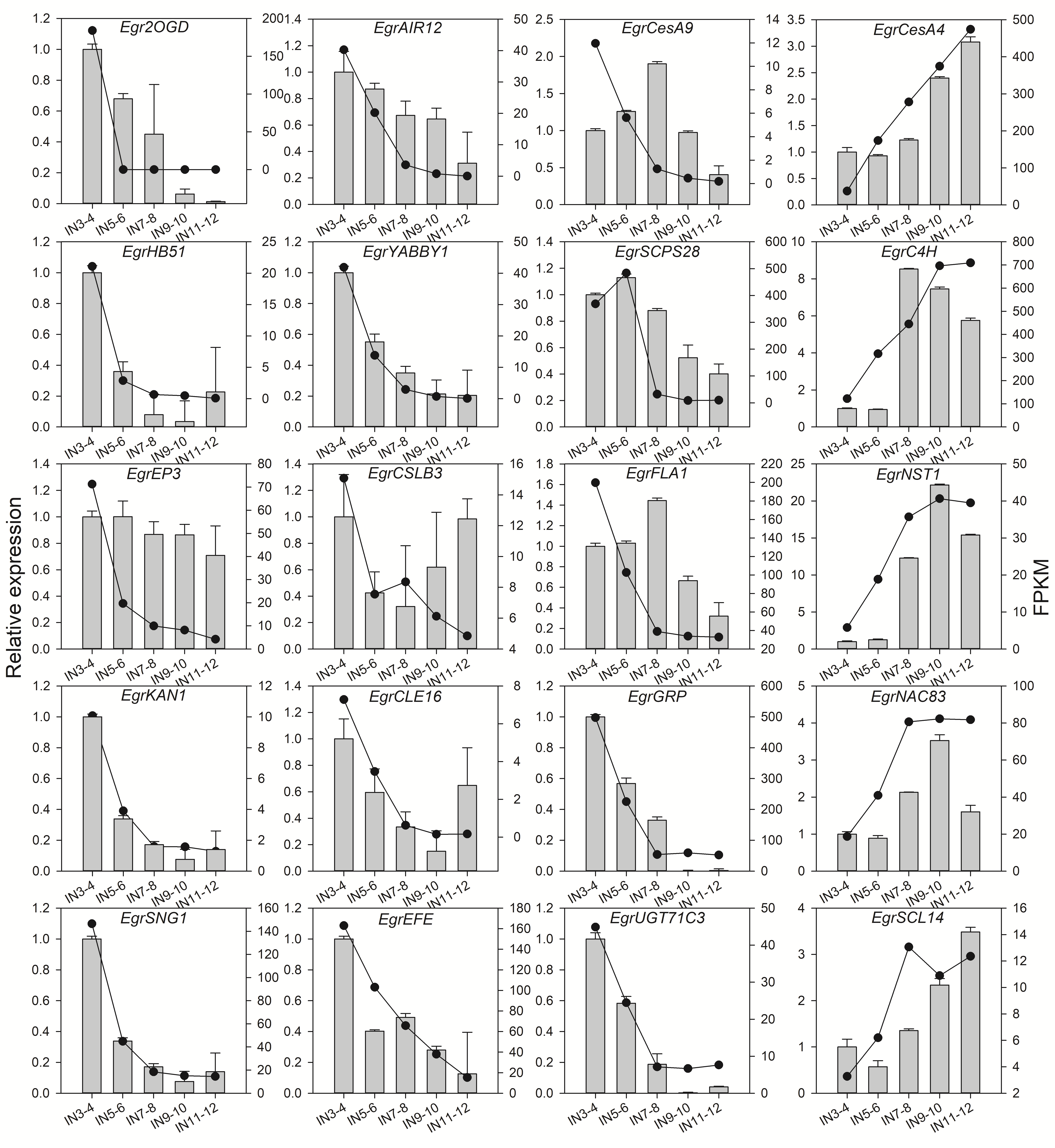

Supplement: Supplementary file 3 — Additional file 3: Supplemental Figure 2. The heat-map of turquoise module genes and the heat map of 70 selected genes from the hub genes in turquoise module. The color bar indicates expression and correlation levels from low(blue) to high (red). Panels IN3, IN5, IN7, IN9 and IN11 represent the different internodes stage, respectively. [file 12870_2024_4731_MOESM3_ESM.tif]

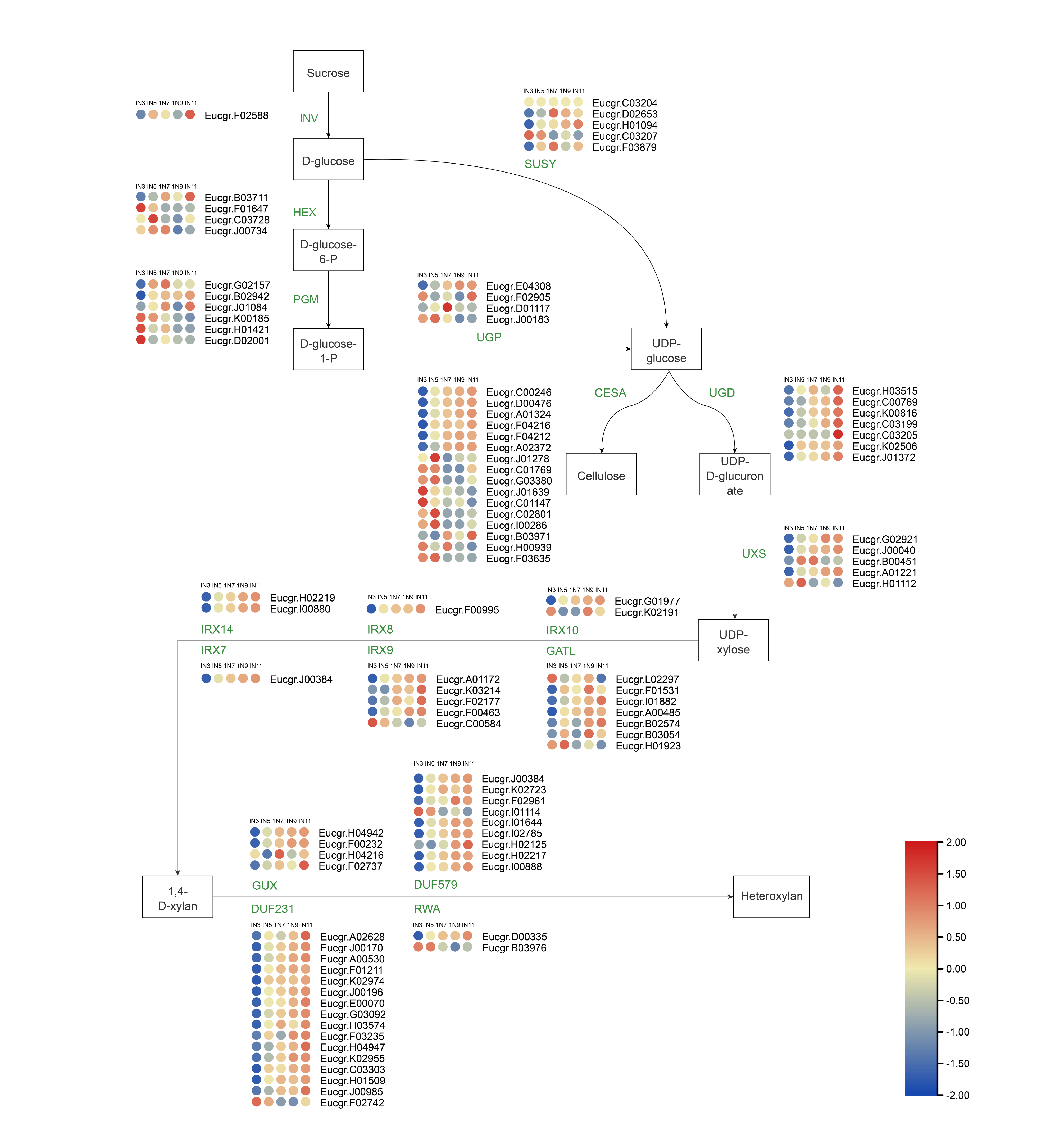

Supplement: Supplementary file 4 — Additional file 4: Supplemental Figure 3. Cellulose synthesis pathway map. The color bar indicates expression and correlation levels from low(blue) to high (red). Panels IN3, IN5, IN7, IN9 and IN11 represent the different internodes stage, respectively. The necessary copyright authorization was secured to use the KEGG image shown in the figure. [file 12870_2024_4731_MOESM4_ESM.tif]
